# Integrated cellular 4D-TIMS lipidomics and transcriptomics for characterization of anti-inflammatory and anti-atherosclerotic phenotype of MyD88-KO macrophages

**DOI:** 10.3389/fcell.2024.1450971

**Published:** 2024-08-23

**Authors:** Carla del Barrio Calvo, Laura Bindila

**Affiliations:** Clinical Lipidomics Unit, Institute of Physiological Chemistry, University Medical Center, Mainz, Germany

**Keywords:** 4D-lipidomics, cell lipidomics, cell transcriptomics, multi-omics, ether lipids, MyD88, atherosclerosis

## Abstract

**Introduction:** Recent progress in cell isolation technologies and high-end omic technologies has allowed investigation of single cell sets across multiple omic domains and a thorough exploration of cellular function and various functional stages. While most multi-omic studies focused on dual RNA and protein analysis of single cell population, it is crucial to include lipid and metabolite profiling to comprehensively elucidate molecular mechanisms and pathways governing cell function, as well as phenotype at different functional stages.

**Methods:** To address this gap, a cellular lipidomics and transcriptomics phenotyping approach employing simultaneous extraction of lipids, metabolites, and RNA from single cell populations combined with untargeted cellular 4 dimensional (4D)-lipidomics profiling along with RNA sequencing was developed to enable comprehensive multi-omic molecular profiling from the lowest possible number of cells. Reference cell models were utilized to determine the minimum number of cells required for this multi-omics analysis. To demonstrate the feasibility of higher resolution cellular multi-omics in early-stage identification of cellular phenotype changes in pathological and physiological conditions we implemented this approach for phenotyping of macrophages in two different activation stages: MyD88-knockout macrophages as a cellular model for atherosclerosis protection, and wild type macrophages.

**Results and Discussion:** This multi-omic study enabled the determination of the lipid content remodeling in macrophages with anti-inflammatory and atherosclerotic protective function acquired by MyD88-KO, hence expedites the understanding of the molecular mechanisms behind immune cells effector functionality and of possible molecular targets for therapeutic intervention. An enriched functional role of phosphatidylcholine and plasmenyl/plasmalogens was shown here to accompany genetic changes underlying macrophages acquisition of anti-inflammatory function, finding that can serve as reference for macrophages reprogramming studies and for general immune and inflammation response to diseases.

## 1 Introduction

As minimal functional and structural units organized by complex hierarchical interactions in multicellular organisms cells and cell’s functionalities are defined by the overlapping role of genome, epigenome, transcriptome, proteome, and metabolome (Vandereyken et al., 2023). Additionally, cells are embedded in an environment led by autocrine, paracrine and endocrine factors that act through ligand-receptor and other interactions to create networks among cell populations. New findings indicate that individual cells display different metabolic characteristics within a clonal population (Yuan et al., 2017). Thereby, the study of the different omics layers contributing to the phenotype and function of cells, at high cellular population resolution, i.e., single-to-low cell numbers, are imperative to address complex questions in translational biology and to understand the molecular circuits underlying cell function. For instance, the holistic view of single cell multi-omic studies can constitute a powerful tool in systems biology, by correlating specific molecular information from single cells to cell populations, tissues, or even organisms. Multi-extraction protocols are becoming increasingly important for therapeutic target discovery due to their ability to capture a wider range of biomolecules. These protocols employ a combination of techniques to maximize the diversity of biomolecules extracted from a single sample. Biological processes rarely operate in isolation, often involving intricate interactions between various biomolecules, including DNA, RNA, proteins, lipids, and metabolites. Multi-extraction protocols allow researchers to study the correlations, networks, and pathways that are dysregulated in disease, in a more holistic manner. Additionally, the broader molecular coverage offered by these protocols increases the chances of discovering novel biomarkers. Multi-extraction protocols also open the door to personalised medicine, as the understanding of an individual’s unique molecular makeup, such as variations in their genes, proteins, and metabolites, can enable treatments tailored to their specific needs, maximizing efficacy and minimizing adverse effects (Boggi et al., 2024). Previously, our lab developed an integrated lipidomics and transcriptomics strategy for low tissue amounts and successfully applied it for investigation of lipidome and transcriptome of functional brain regions and subregions. The dual lipid/RNA extraction was combined with targeted mass spectrometry analysis and qPCR to enable sensitive quantitative profiling of specific lipid and RNA targets relevant to epileptic seizures in mouse models (Lerner et al., 2018; Lerner et al., 2019; Post et al., 2022).

The latest advances in cell isolation together with high-end analytical techniques and new computational tools for data processing and integration, allow multi-omic investigations for molecular profiling of single cells and also the collection and analysis of large-scale data from different omics (Zhu et al., 2020; Watson et al., 2022). Building on this, single cell studies comprising multiple layers of molecular information, unravel cell-to-cell heterogeneity and stochasticity, whereas during bulk cellular analysis, variability is eliminated by averaging, masking molecular signatures of individual cells and leading to biased conclusions. Primary emphasis of single cell multi-omic analysis lies on the integration of DNA and RNA or DNA/RNA and protein data, allowing for disease subtyping based on DNA and/or RNA data, and a more detailed molecular insight provided by the proteomic dimension. Unlike proteome, genome, or transcriptome, which are also governed by regulatory mechanisms involving post-translational modifications of proteins and epigenetic regulation, the capture of cell dynamics, real-time biochemical depiction, and ultimate downstream biochemical products essential for phenotype association can only be accomplished through the integration of metabolomics and lipidomics. This is because, lipidome and metabolome composition varies substantially during various cellular differentiation, proliferation, and reprogramming states and across cell populations. (Lee et al., 2020). Multi-omics analyses at the cellular level are crucial for elucidating the complexities of cellular biology. For instance, in-depth molecular characterization enabled by cellular multi-omics helps define the unique properties of each cellular population, including surface markers, gene expression patterns, protein abundance, and metabolic activity, as well as their functional diversity. This knowledge is essential for advancing our understanding of development, disease, and personalized medicine (Liang et al., 2024). Hence, there is a pressing need for the combined analysis of transcriptomics, lipidomics and metabolomics in single cell and cell subset studies (Capolupo et al., 2022).

Increasing evidence suggests that abnormal cellular metabolism, including lipid dysfunctions, of immune and non-immune cells is connected to abnormalities in the immune response. The immune system plays an important role in inflammation, which is linked to various chronic disorders such as obesity and diabetes, cardiovascular diseases, cancer, neurodegenerative and metabolic diseases (Zhang et al., 2022). The same is true for pro-inflammatory signaling molecules, which interfere in lipid metabolism. Despite the evident crosstalk between lipid metabolism, inflammation and health, the molecular pathways and lipid function underlying these pathological and physiological conditions are still little-known. In this regard, higher resolution cellular multi-omics can be a promising venue to accelerate early-stage identification of cellular phenotype changes in disease conditions and the subsequent determination molecular and pathway targets for therapeutic intervention (Yu et al., 2014).

Modern mass spectrometric technologies coupled with high resolution ion mobility (IMS-MS) enable higher structural resolution and possibly sensitivity allowing in-depth molecular profiling of small biological specimens (Burnum-Johnson et al., 2019). While MS allows for the separation of ions based on their mass-to-charge (*m/z*), ion mobility enables gas-phase separation of ionized organic molecules by their collisional cross section (CCS), which is inherently dependent on conformation, charge, as well as mass. This extra dimension of ion separation can increase sensitivity of individual species detection and peak sampling capabilities, making IMS-MS a robust analytical tool to elucidate chemical structure and separate complex mixtures (Kanu et al., 2008). The high ion utilization efficiency of trapped ion mobility spectrometry (TIMS) along with a novel MS scan mode called parallel accumulation-serial fragmentation (PASEF) make it an enticing platform for in-depth and sensitive qualitative and quantitative molecular profiling particularly for lipidomics and proteomics. (Meier et al., 2018; Paglia et al., 2022; Bennett et al., 2023; Lerner et al., 2023; Mayer and Karl, 2023; Shapiro and Bassani-Sternberg, 2023; Merciai et al., 2024). Multi-omic analysis for disease marker identification and better understanding of disease mechanisms has become an essential approach in biomedical research. Through multi-omic cellular approaches, researchers can elucidate the molecular pathways and regulatory networks and identify novel targets and biomarkers for the diagnosis, treatment, and prevention of different chronic diseases (Liang et al., 2024). Accordingly, we set out to develop an integrated lipidomics and transcriptomic protocol for cellular profiling, amenable for high lipidome and transcriptome coverage from cell subsets and single cell populations of low number of cells. We subsequently applied this approach for the characterization of the lipid and RNA changes and pathways associated with an anti-inflammatory and anti-atherosclerosis macrophage phenotype of MYD 88 KO.

Atherosclerosis is a chronic condition that affects the arteries and is linked to systemic inflammation. It is responsible for about fifty percent of all deaths in westernized societies. A thorough understanding of the cell-specific signalling mechanisms that mediate the inflammatory response is crucial for improving anti-inflammatory therapies and reducing mortality and morbidity (Ridker and Thomas, 2014). Atherosclerosis involves the dysregulation of macrophages due to uptake of modified lipids, formation of cholesterol crystals, and lipid and inflammatory mediators that favour foam cell formation. This also affects monocytes and leads to different states of macrophage activation with both pro- and anti-inflammatory phenotypes (Poznyak et al., 2021). MyD88 (myeloid differentiation primary response 88) is an adaptor protein that plays a significant role in initiating and amplifying the immune response in atherosclerosis by inducing signalling from multiple receptors at the plasma membrane and endosomes (Akira 2003; Podrez et al., 2002; Ishii et al., 1996; Ma et al., 2022). MyD88 signalling can trigger production of pro- or anti-inflammatory cytokines as well as the activation of other inflammatory factor such as type I IFNs, NF-kB and AP-1 through various receptors including TLRs and several cytokine receptors that are associated to the ability of macrophages to polarize toward the M1 phenotype. M1 macrophages constitute the most common cell population in lesions of patients with coronary heart disease. While this pathway was initially characterized in innate cells, it has been found that MyD88 is broadly expressed across most cell types of the immune system and cardiovascular systems, often exerting distinct roles specific to certain cell types within cardiovascular disease contexts (Blagov et al., 2023). While its role during pathogenic responses is well understood, new insights into molecular mechanisms underlying inflammatory responses in atherosclerosis are emerging, providing valuable insights for potential therapeutic targets. MyD88 knockout macrophages have demonstrated reduced plaque formation indicating their potential use for studying atherosclerosis protection. These findings highlight targeting MyD88 signalling in macrophages as a promising approach for reducing inflammation and protecting against atherosclerosis (Bayer and Alcaide, 2021). The widespread involvement of these pathways in cardiovascular endurance is the basis for future mechanistic studies which may identify MyD88 as effective target for therapeutic intervention in cardiovascular diseases (Akira, 2003). In this study we set out to characterize in-depth how the lipid composition changes and which lipid pathways are effected in macrophages upon acquiring an anti-inflammatory phenotype due to MyD88 knock-out gene. Additionally, the interplay with the transcriptome changes is expected to expand the window of understanding of the anti-atherosclerotic and anti-inflammatory function of macrophages and to serve as a reference for future studies of macrophages and immune cell reprogramming and function. Specifically, we aimed to uncover potential molecular mechanisms and fingerprints related to atherosclerosis protection of MYD 88 deficient macrophages. To this end, we developed and applied a dual extraction approach of cellular lipidome and transcriptome and combined it with high-end 4D-TIMS lipidomics and RNA sequencing in order to, for the first time due to our knowledge, comprehensively investigate the lipidome, of more than 400 lipids, and the transcriptome of MyD88-KO macrophages. The dual-extraction protocol of lipidome and transcriptome from a single cellular population of low number of cells, presented in this article, combined with the cellular 4D-TIMS lipidomics and RNA sequencing, enable uncovering of lipids, RNA, and their interplay and associations with diseases and identification of new potential disease-specific fingerprints and integrated pathways, not accessible through single-extraction methods.

## 2 Materials and methods

### 2.1 Samples

Human Embryonic Kidney (HEK) 293 cells were obtained from the Clinical Lipidomics Unit and Institute of Physiological Chemistry of the University Medical Center of Mainz, Germany. Macrophages MyD88-KO (ENH179-FP) and CT (ENH167-FP) were purchased from Kerafast (Shirley, United States). In-house existing mouse brain tissue was used as a proxy to multicellular biological sample in the first steps of 4-Dimensional (4D) trapped ion mobility mass spectrometry-(tims) development for cellular profiling and in assessment of amenability of dual lipidomics and transcriptomic extraction for subsequent unbiased lipidome analysis at high coverage (Lerner et al., 2018; Post et al., 2022).

### 2.2 Co-extraction and analysis of lipids, metabolites, and RNA in cells

Lipids and metabolites extraction from reference cells and tissues was carried out using a classical liquid-liquid extraction (LLE) technique, utilizing methyl tert-butyl ether (MTBE)/methanol (MeOH) (10:3; v/v) and 0.1% formic acid (FA) to separate non-polar and polar compounds into distinct phases. This extraction was used to: i) assess and tailor analytical and processing parameters for 4D-cellular lipidomics that are conducive to high coverage of lipidome in complex cellular matrices, e.g., cells and tissue sample as a proxy to multicellular biospecimens. For the latter, in-house available brain tissue was used; ii) evaluate the lipidome coverage following co-extraction of lipids and RNA and compare it with the lipidome coverage obtained after classical lipid LLE extraction, iii) establish an initial spectral library, as well as RT and CCS reference values of cellular lipidome and multicellular lipidome using brain tissue as a proxy. This cellular spectral library and annotation parameters for 4D-TIMS analysis was curated by manual annotation of lipid identities and structures, collisional cross section values, retention times and fragmentation patterns, using MS-Dial data bases and our previously established 4D-libraries from lipid standards and plasma lipids.

**FIGURE 1:**
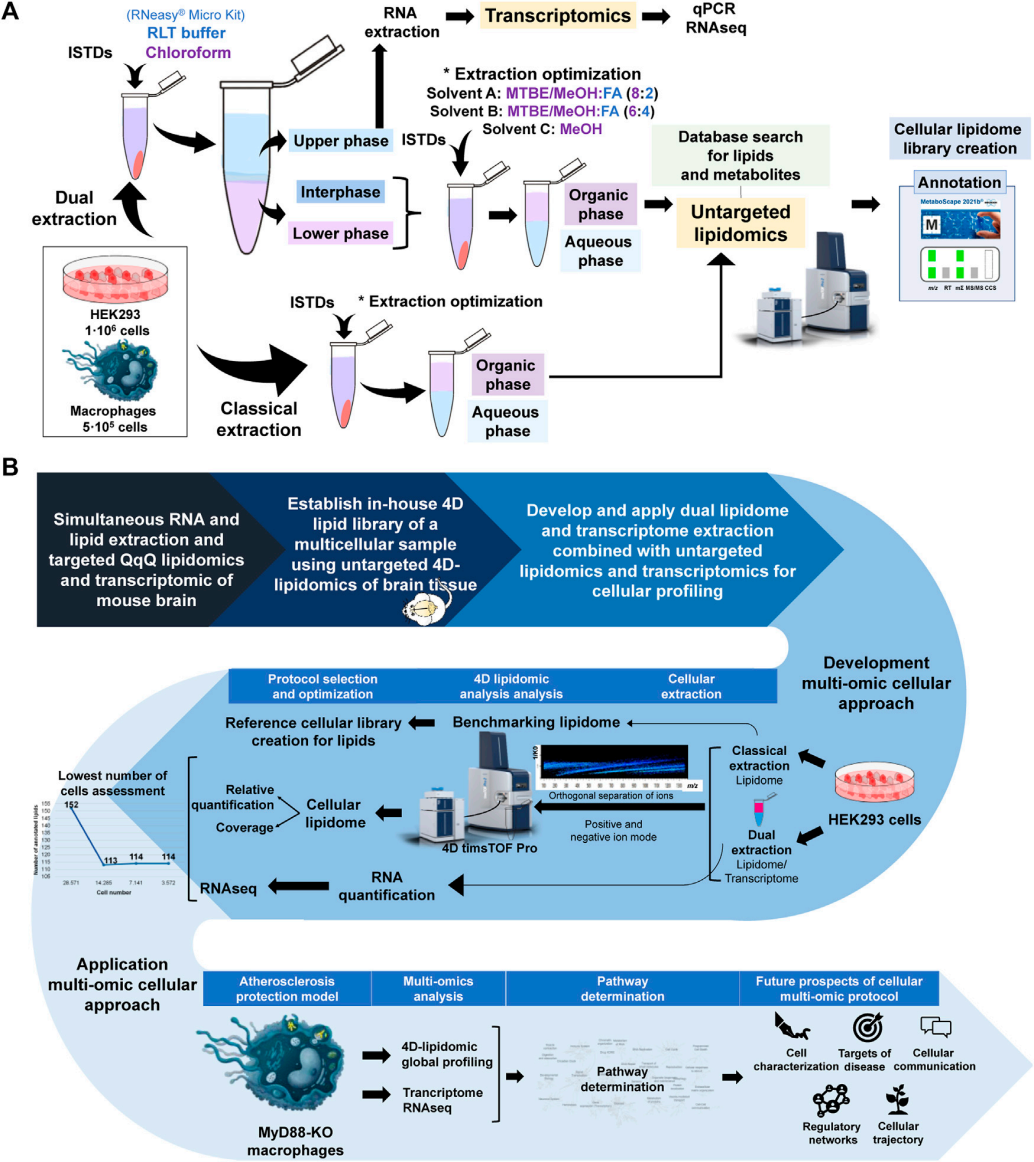
**(A)** Schematic of experimental workflow. UHPLC Elute LC system and TIMSTOF Pro MS are reproduced from Bruker (Bremen, Germany). **(B)** Schematic display of the rationale of study design, analytical workflow and application of the cellular lipidomics and transcriptomics approach for tha characterization of the MYD88 KO macrophages. UHPLC Elute LC system and TIMSTOF Pro MS are reproduced from Bruker (Bremen, Germany).

Lerner et al. improved the traditional liquid-liquid extraction method of tissues by integrating RNA co-extraction using the RNeasy^®^ Mini Kit, enabling dual extraction of lipids and RNA from the same tissue sample and subsequent analysis via a targeted lipidomic approach (Lerner et al., 2018; Post et al., 2022). Essentially, chloroform and RNeasy^®^ Micro Kit buffer along with internal standards were added to the sample prior to extraction of RNA and subsequent MTBE-based LLE extraction of lipids. Building upon this prior dual extraction protocol, we further optimized the dual extraction protocol to achieve cell-level resolution using LLE strategy and the RNeasy^®^ Micro Kit for RNA extraction, while also evaluating the effectiveness of the sample preparation and extraction procedure for high coverage lipidome by untargeted 4D-TIMS lipidomics. Internal standards mixture was prepared in 200 μL of MeOH and added to the cell pellet together with the extraction solvent. The cell solution is further homogenized using Precellys^®^ (5,000 rpm, 15 s) to ensure its efficient disruption and subsequently centrifuged at maximum speed for 5 min, to separate the upper aqueous phase and the lower organic phase. Different solvent ratios were tested for 1 million HEK293 cells for the lower organic phase (A, MTBE:MeOH (10:3; v/v)/FA (0.1 M) in a proportion of 8/2; B, MTBE:MeOH (10:3; v/v)/FA (0.1 M) in a proportion of 6/4 and; C, MeOH). The lipid extract was obtained after vortexing (45 min, 4 °C) and centrifugation at (15 min, 1,300 g). Classical extraction of lipids using LLE method from HEK293 cells was used to compare the performance of dual lipidome/transcriptome extraction. For this, 1 million HEK293 cells was homogenized with Precellys^®^ (5,000 rpm, 15 s) and centrifugated at maximum speed for 5 min, after the addition of internal standards and extraction solvent. To evaluate linearity and lowest number of cells from which lipidome and transcriptome is analyzable, 1,000,000, 500,000, 250,000, 125,000, 62,500, 31,250, 15,625, 7,813 and 3,906 cells, respectively, were extracted and analyzed using both extraction protocols. All lipid extracts were evaporated and stored at −20°C till further analysis. The aqueous phases obtained from the dual extraction protocol were immediately processed for RNA extraction using RNeasy^®^ Micro Kit protocol. The lipid extracts were resolubilized in MeOH/H_2_O (9:1; v/v) for 4D lipidomics analysis. 4D-TIMS lipidomics and Metaboscape 2021b (Bruker, Bremen Germany) with in-house created 4D-lipid cellular library and/or Metabobase, for metabolite identification, was utilized for the subsequent lipidomic and metabolomic investigation (Figure 1).

#### 2.2.1 Lipid deuterated and non-deuterated internal standards

Deuterated and non-deuterated internal standards (ISTDs) from Avanti Polar Lipids, Inc., USA, were used for relative quantification (see Supplementary Material).

#### 2.2.2 Chemicals and reagents

The following LC-MS grade solvents and reagents used in analytical workflow were purchased from Merck (Germany): chloroform water, methanol (MeOH), 2-propanol, formic acid (FA), triethylamine, ammonium formate, acetonitrile (ACN) and methyl tert-butyl ether (MTBE). Absolute ethanol was purchased from Honeywell (North Carolina, United States). The RNeasy^®^ Micro Kit was purchased from QIAGEN (Venlo, Netherlands).

#### 2.2.3 Untargeted 4-dimensional (4D) trapped ion mobility mass spectrometry (TIMS) cellular lipidomics

For lipid separation, an Elute UHPLC system (Bruker Daltonics, Bremen, Germany) with a C18 Luna Omega column (100 Å × 2.1 mm × 1.6 μm) purchased from Phenomenex (Germany) was used to perform the reversed phase (RP) chromatographic separation of samples. The column was thermostated at 45 °C. The separation solvent and gradient system used for the lipidomic and metabolomic approach in negative and positive ion mode as well as the liquid chromatography (LC) lipidomic gradient is the same as described by Lerner et al. (2023). This gradient was run at a flow rate of 0.2 mL/min. In positive mode, the injection volume onto the column was 10 μL, whereas in negative mode, it was 20 μL. Throughout the analysis, the auto-sampler remained consistently at 4°C. The experiments were conducted in a hybrid TIMS-qToF mass spectrometer coupled to an Elute UHPLC using a Bruker Daltonics TIMS-qToF pro instrument (Bruker Daltonics, Germany) for both negative ion mode and positive ion mode. For fragmentation analysis, the scan mode was set to PASEF with the mass scan range of 100–1,350 Da for both MS and MS2 acquisition. The acquisition cycle consisted of 0.1 s with the mobility scan range of 0.55–1.87 V*s/cm2 for the positive mode and 0.55–1.86 V*s/cm2 for the negative mode. Both the TIMS and mass calibration of the instrument was carried out on a weekly basis with the following peaks from the Agilent ESI LC-MS tuning mix [m/z, 1/K0: (322.0481, 0.7318 Vs. cm−2), (622.0289, 0.9848 Vs. cm−2), (922.0097, 1.1895 Vs. cm−2), (1,221.9906, 1.3820 Vs. cm−2)] in the positive mode, and [m/z, 1/K0: (666.01879, 1.0371 Vs. cm−2), (965.9996, 1.2255 Vs. cm−2), (1,265.9809, 1.3785 Vs. cm−2)] in the negative mode. The parameters utilized in these experiments are consistent with those described by Lerner et al. (2023).

#### 2.2.4 Transcriptomics

RNA validation and quantification was performed using a Nanodrop 2000/2000c spectrophotometer (Thermo Fisher Scientific, Germany). RNA integrity number (RIN) and RNAseq were performed using Agilent 2100 Bioanalyzer System in Starseq facility at Johann-Joachim-Becher-Weg 30a (D-55099) Mainz, Germany.

### 2.3 Data and statistical analysis and pathway determination

Compass Hystar 6.2 direct the LC instrument, while timsControl 2 (Bruker Daltonics in Bremen, Germany) is used to control and monitors the TIMS-TOF instrument’s instrumental calibration and data collection. Compass DataAnalysis and Metaboscape 2021b, both from Bremen, Germany’s Bruker Daltonics, were used for processing and extraction of the lipid features, quality control assessment, annotation and curation of lipid data, respectively. Metaboscape 2021b was used for extraction of the 4 dimensional (4D) features, peak area of individual signals, lipid identification and curation, spectral library establishment. Compass DataAnalysis was used to retrieve ion mobility and extracted ion chromatogram for lipid data curation and verification, fragmentation spectra inspection in individual samples, and general data visualisation for annotation and quality control purposes. (Lerner et al., 2023).

GraphPad Software (Boston, United States) and Origin (OriginLab Corporation, Northampton, United States) were used for statistical analysis. Lipid Pathway Enrichment Analysis (LIPEA, https://hyperlipea.org/home), created by Biomedical Cybernetics Group (Dresden, Germany) and Reactome (http://www.reactome.org), a curated and peer-reviewed resource of human biological processes, were used for pathway discovery.

## 3 Results

### 3.1 Development of dual lipidomic and transcriptomic methods for cells

#### 3.1.1 Assessment of 4D-TIMS lipidomics profiling method for cellular lipidome and metabolome

First, we assessed the effectiveness of the untargeted 4D LC-TIMS-PASEF-MS for the comprehensive analysis of cellular lipids and metabolites. To this end, untargeted 4D-TIMS analysis of a brain tissue lipidome, extracted using classical lipid extraction was performed to ascertain the suitability of 4D-TIMS lipidomics from a complex multicellular matrix and establish an extended panel of 4D lipid library to be used for initial annotation of cellular lipidome. Similarly, lipid extracts of 1 million HEK293 cells and subsequent 4D-TIMS-PASEF analysis was performed. HEK293 cellular lipidome was annotated using the in-house existing 4D-libraries for tissue, lipid standards, MS-DIAL, and manual annotation. (Lerner et al., 2023). Additionally, metabolites were annotated with Bruker Metabobase spectral library. These results laid the foundation for creating an initial 4D-cellular reference lipid library which will aid in identification of lipid structures in subsequent stages of development including for macrophage lipidome annotation.

**FIGURE 2:**
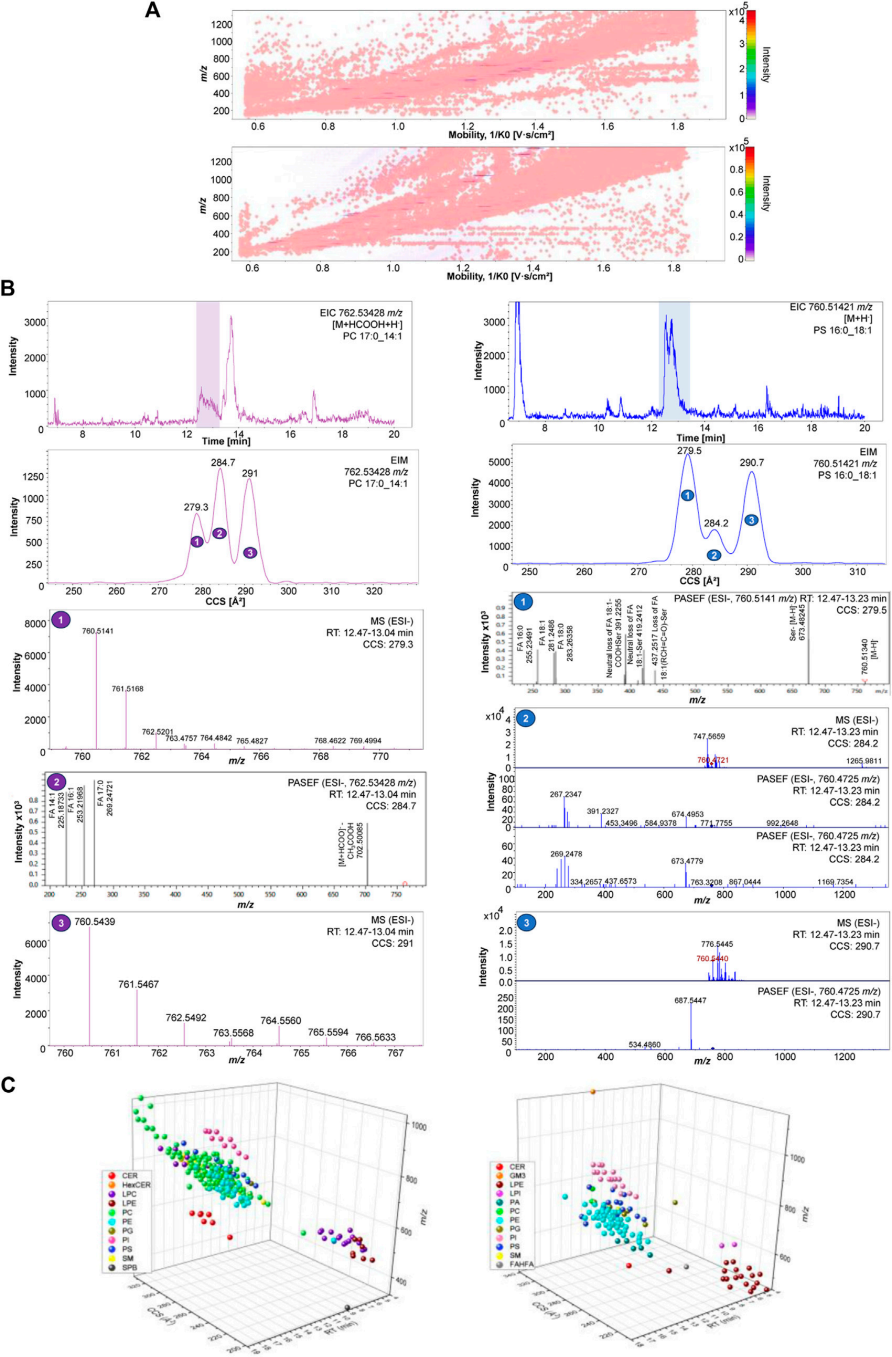
**(A)** 2D-hystogram of cellular lipidome distribution of raw data in 2D mobility and *m/z* dimension of ions using ESI+ (left) and ESI- (right). **(B)** PC 17:0_14:1 762.53428 *m/z* EIC, EIM, MS and MS/MS in ESI- (left); PS 16:0_18:1 760.51421 *m/z* EIC, EIM, MS and MS/MS in ESI- (right). **(C)** 3D-scatter plot cellular lipidome separation across lipid classes using using ESI+ (left) and ESI- (right).

This approach illustrates the potential of combining mass spectrometry and ion mobility with a UHPLC microliter flow for orthogonal separation of molecules in this cellular matrix (Figure 2A). The advantage of orthogonal lipid separation by microliter flow RP-UHPLC chromatography combined with TIMS-PASEF for cellular lipids analysis is evident in Figure 2B, where mobility-based separation of PC 17:0_14:1 and PS 16:0_18:1 isomers compensates the chromatographic separation and allows delineation of the compositional and/or possible configurational isomers. The EIM frames, as shown in Figure 2B, demonstrates how ions are separated in this additional mobility dimension, enabling discovery, separation, and identification of molecules. Accordingly, the EIM results indicate that ion mobility introduces a new separation dimension facilitating peak-based separation of isomers for both phospholipid species. The MS and MS/MS of each mobility frame are named from 1 to 3, from the lowest to the highest CCS value, respectively. The MS/MS spectra obtained for the second mobility frame in Figure 2B (left) show the diagnostic fragments corresponding to PC 17:0_14:1, whereas in Figure 2B (right) diagnostic fragments corresponding to PS 16:0_18:1, as well as the FA 18:0 corresponding to another lipid specie are detected. Although MS/MS from the precursor ions at *m/z* 762.53428 in Figure 2B were only obtained for the second mobility frame, IM demonstrates its potential in reducing background noise and revealing new characteristic CCS features of each ion, allowing thus identification and discovery of lipids that would not be possible with TIMS off. Figure 2C depicts how the values of *m/z*, RT, and CCS contribute to the spatial separation of lipids within their respective classes (Supplementary Material). It is evident that CCS plays a significant role in determining the spatial distribution of lipids, enhancing peak resolution and confidence of structural identification. Therefore, with this strategy we are able not only to enhance the characterization and differentiation of complex mixtures, but also to provide valuable insights into chemical structure and composition.

#### 3.1.2 Optimization of dual cellular lipidome extraction in HEK293 cells

The previous assessment steps have all contributed to ascertain the advantages of 4D-TIMS- cellular lipidome for facilitating high coverage of lipidome and high-throughput profiling, which are necessary for integrating cellular multi-omic approaches. We, hence focused further on assessing, using reference HEK293 cells, the amenability of dual cellular lipidome and transcriptome extraction for subsequent comprehensive 4D-cellular lipidome profiling and RNA sequencing. Comparative coverage of the lipidome using 4D-TIMS lipidomics was performed to ascertain performance of classical and dual extraction methods for high lipidome coverage and quantification.

**FIGURE 3:**
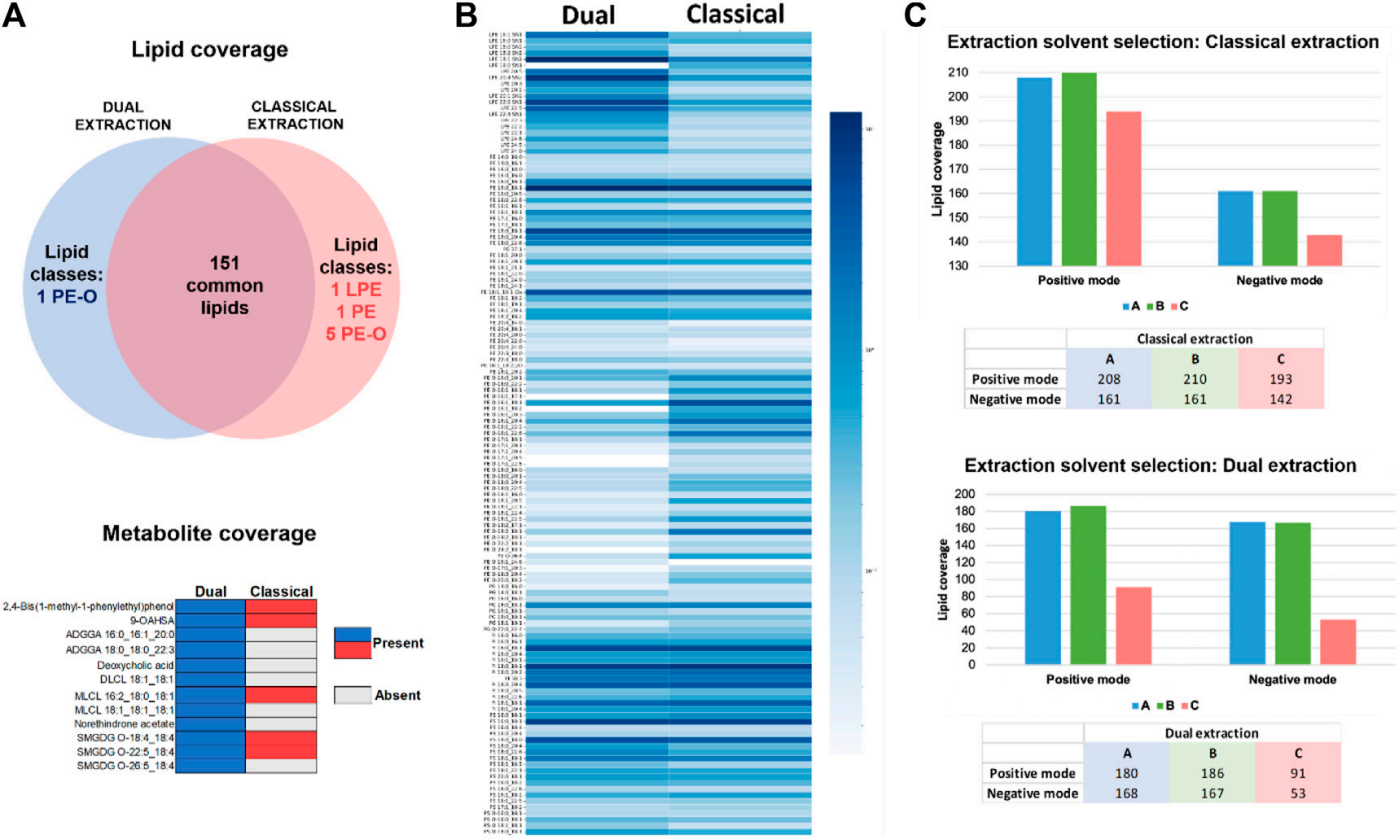
**(A)** Venn diagram representation of specific and common lipids to classical and dual extraction methods in ESI- (up). Metabolites presence and absence in classical versus dual extraction methods in ESI- (down). **(B)** Lipid quantification comparison of dual vs. classical method in ESI-. Log_10_ of the quantified values was used for the visualization. Color grade from dark to light blue indicating each lipid specie abundance. **(C)** Lipid coverage comparison for three different solvents using the classical extraction in ESI+ and ESI- (upper graph) and dual extraction in ESI+ and ESI- (bottom graph).

Lipids extracted using the previously developed dual lipidomic & transcriptomic protocol and the classical lipid extraction were analyzed in negative mode within a single batch and simultaneously processed (annotation and relative quantification) in Metaboscape to better compare the lipidome coverage using both methods. While 7 lipids were identified specifically using the classical extraction and 1 lipid was present only when the dual extraction was applied, 151 lipids were commonly identified with both extraction methods (Figure 3). The metabolite interrogation using Metabobase annotation in both extracts demonstrates the superior effectiveness of the dual extraction in profiling the metabolome of this cell model. Although only 15 metabolites were detected using dual lipidomic & transcriptomic extraction compared to 6 metabolites using the classical extraction (Figure 3) it suggests that prospective use of an appropriate metabolomic platform can allow the co-investigation of low mass metabolites and provision of additional valuable information on metabolome. Based on the results obtained for the 4D-lipidome of HEK293 cells, two spectral libraries were created for positive and negative ion modes, named: “Multi-omic-derived cellular Lipidome 2023 pos” and “Multi-omic-derived cellular Lipidome 2023 neg”, respectively.

The comparison of lipid levels between the classical and dual extraction methods in negative ion mode (Figure 3) evidence that the dual extraction method yields higher lipid levels for certain lipid classes compared to the classical method Specifically, LPE, PS, and PS-O exhibit higher quantification values when the dual extraction strategy is employed. On the other hand, the classical extraction strategy results in higher levels of the PE-O/P lipid class compared to the dual extraction and PE, PG, and PI are equally represented in both extraction methods. Considering the future prospects of this method in single cell multi-omics, where sensitivity holds significant value, the dual extraction procedure was selected as the preferred approach for further analysis in cells. The ability to enable simultaneous analysis of RNA, lipids, and metabolites from the same sample is particularly advantageous in cellular multi-omics research, where limited sample availability and low volumes of cell suspensions or homogenates are frequently encountered and not readily aliquotable for individual omic extractions. Moreover, dual extraction demonstrated comparable lipid coverage and superior metabolite coverage and lipid quantification values for most lipid classes. Aiming at further identifying the optimal solvent composition and solvent ratios for qualifying and quantifying lipids and metabolites in cells using dual extraction, two experiments were conducted in parallel using the classical and dual extraction protocols, respectively. For each extraction, three distinct solvents were employed for 1 million HEK293 cells. The lipid extracts were analyzed using 4D-TIMS untargeted lipidomics and annotated using the established extended lipidome 4D-library (see above). The primary focus was to assess the suitability of extraction procedure for 4D-TIMS cellular lipidomics and metabolomics using a population of 1 million HEK293 cells, with emphasis on lipid and metabolite coverage as well as lipid quantification. Three different extraction solvents were assessed to enhance the yield of lipidomic and metabolomic analyses. (Each extraction method is designated by a letter corresponding to the type and proportion of solvents used): A, MTBE:MeOH (10:3; v/v)/FA (0.1 M) in a proportion of 8/2; B, MTBE:MeOH (10:3; v/v)/FA (0.1 M) in a proportion of 6/4, and; C, MeOH. First, a higher lipid coverage was obtained for solvents A and B compared to C in both negative and positive mode (Figure 3), demonstrating the superiority of MTBE:MeOH/FA over MeOH in terms of lipid coverage. Secondly, upon closer examination of the number of annotated lipids in the MTBE:MeOH/FA fractions, it is observed that B solvent yields slightly higher results in both classical and dual extraction methods (Supplementary Material).

To conclude, similar quantification results were obtained with both classical and dual extractions, demonstrating that the solvents A and B containing MTBE are better suited than methanol as they provide improved quantification values and a higher lipidome coverage, while the HEK293′ characteristic lipidome significantly fades in both experiments and ion modes when MeOH is employed as extraction solvent (Supplementary Figure S1). Hence, it is evident that MTBE-containing solvents render an acceptable lipid coverage using both classical and dual extraction strategies. Solvent B consistently outperforms A solvent in terms of quantitative coverage across various lipid classes. This is evident from the consistently improved quantification coverage of LPE-O and SM, as well as PE, PE-O, PE-P, PC-O, PI-O, and PS species.

#### 3.1.3 Cellular lipidomics, metabolomics and transcriptomics in a low number of cells

**FIGURE 4:**
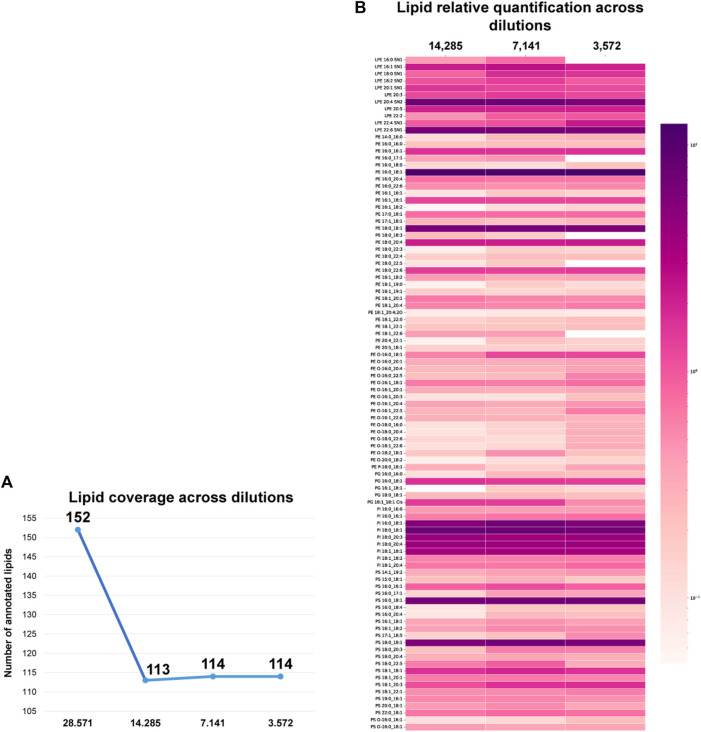
**(A)** Lipid coverage across organic extracts of 28,571, 14,285, 7,141 and 3,572 cells in ESI-. **(B)** Lipid quantification values across different cell numbers in negative ion mode 4D-cellular lipidomics. Log_10_ of the quantified values was used for the visualization. Color grade from dark purple to light pink indicating each lipid specie abundance.

In order to develop a sensitive cellular analytical platform using dual-omic extraction and microliter flow 4D-TIMS cellular lipidomic profiling, two experiments were conducted to achieve comprehensive lipidome profiling at high cellular resolution, i.e., in low cell numbers. In a first experiment, the organic phase previously extracted using dual extraction was serially diluted. In another experiment, the cell suspension of 1 million cells was also serially diluted for subsequent extraction using dual and classical protocols for lipid and RNA analysis. During the initial experiment, the organic phase aliquot corresponding to 28,571 cells was sequentially diluted by factors of 1/2, 1/4, and 1/8 resulting in dilutions corresponding to 14,285, 7,141, and finally down to 3,572 cells respectively. The diluted lipid extracts were analyzed in negative ion mode and annotated with the extended 4D-cellular lipidome library. Supplementary Figure S2 depicts the peak area linearity, e.g., R-squared (R^2^) for different lipids across dilutions. The analysis reveals that a significant portion of the annotated lipids (78.07%) exhibit an R^2^ value ranging from 0.8 to 1, evidencing the alignment of our results with a linear regression across different dilutions. Only 21.92% of the annotated lipids exhibit an R^2^ below 0.8. The robustness of 4D-TIMS lipidomics and dual omic extraction for low cell numbers profiling is illustrated in Figure 4, where the lipid coverage across extracts of different cell numbers is represented. When comparing the diluted extracts, a significant decrease, i.e., from 152 to 113 in the number of identified lipids is observed between 28,571 and 14,285 cells, followed by a rather steady coverage with, i.e. 114, and 109 lipids for subsequent dilutions, respectively. It is obvious that the sensitivity of lipid detection reaches a steady threshold from about 14.000 cells downwards. This indicates, however, the robustness of the approach in terms of maintaining consistent the lipid coverage in cellular lipidomics in a low number of cells, but also the lowest threshold of cell number at which with current analytical conditions cellular lipidome can be reliably quantified. We consider, however, that the 113, 114 and 109 number of identified lipids across the three lowest dilutions points in negative ion mode is sufficiently informative of researching the cellular lipidome content and function as it contains most of the representative and specific species of each lipid class. The method’s quantitative performance for low cell numbers is illustrated in Figure 4 where the normalized lipid values (normalized to internal standards) across the different dilutions are compared. Remarkably, the relative quantification values remain consistent across the various cell dilutions, including the highest dilution, highlighting the method’s ability to reliably quantify lipids even in limited cell amounts and for low abundant lipid species as well. Only a few lipid species are absent in the extract of 3,572 cells, indicating a good limit of detection and quantification of a specific set of lipids in samples with as low as 3,572 cells.

**FIGURE 5:**
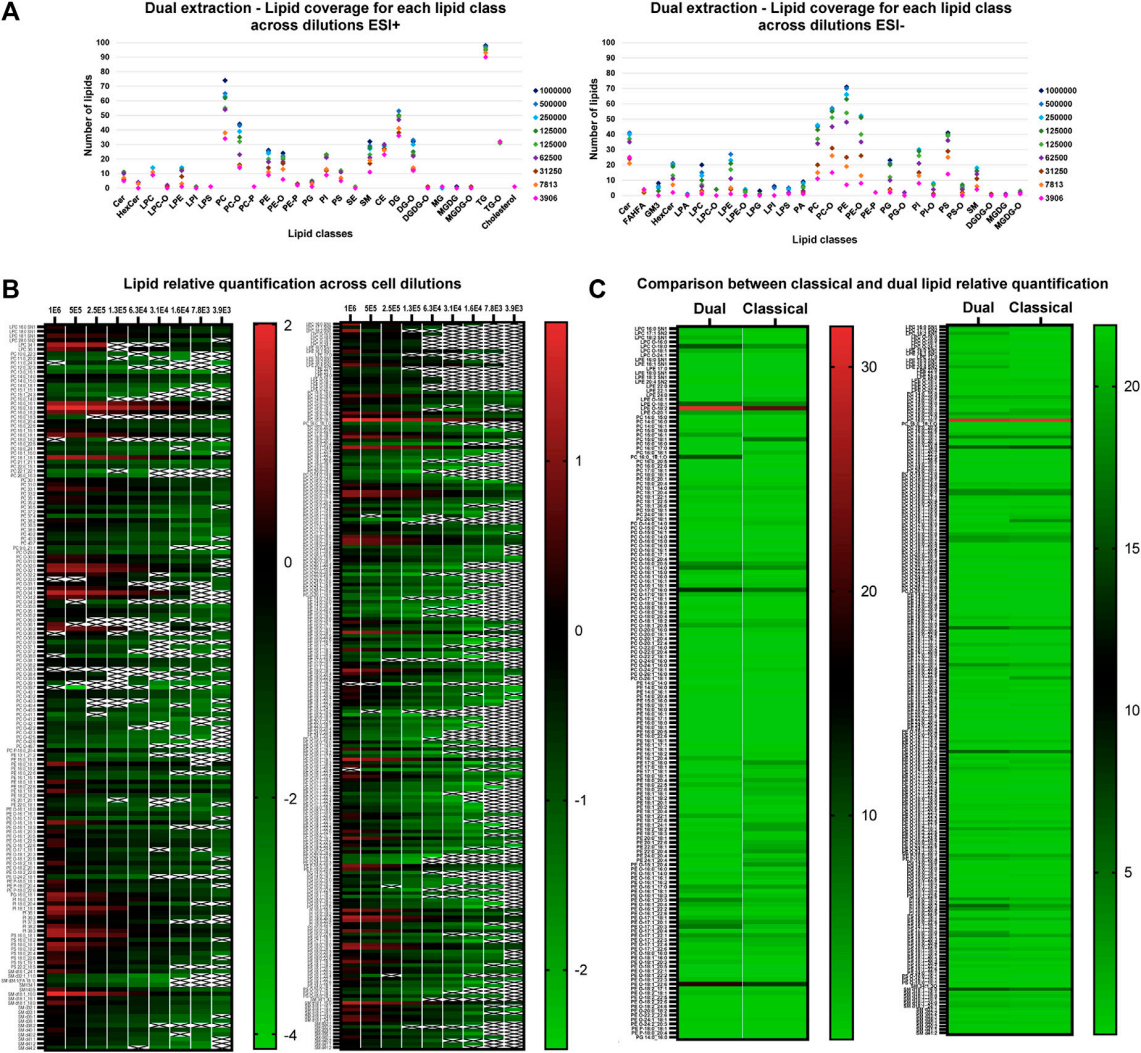
**(A)** Lipid coverage in positive (left) and negative ion mode (right) by 4D-TIMS lipidomics across different cell number dilutions (1,000,000, 500,000, 250,000, 125,000, 62,500, 31,250, 15,625, 7,813 and 3,906 cells) via optimized dual lipidome and transcriptome extraction. **(B)** Lipid relative log_10_ quantification values across cell dilutions (1,000,000, 500,000, 250,000, 125,000, 62,500, 31,250, 15,625, 7,813 and 3,906 cells in ESI+ (left) and ESI- (right) using dual extraction. With a “X” represented those samples which were not quantifiable. To simplify, TG, DG and CE were discarded from the analysis. **(C)** Lipid quantification values across cell dilutions in ESI+ (right) and ESI- (left) comparing both extraction methods. To simplify, TG, DG and CE were discarded from the analysis.

Finally, we applied the optimized dual extraction and lipidome analysis on 9 different cell dilutions (1,000,000, 500,000, 250,000, 125,000, 62,500, 31,250, 15,625, 7,813 and 3,906 cells). The lipid extracts of the diluted cell samples were analyzed in negative and positive ion mode and annotated using the 4D-cellular lipidome library, MSDIAL and Bruker Metabobase spectral libraries for lipids and metabolites. Figure 5 displays the lipid coverage, data linearity and reproducibility of identified lipids extracted with optimized dual extraction and analyzed by 4D-TIMS cellular lipidomics covering 9 cell number dilutions (1,000,000, 500,000, 250,000, 125,000, 62,500, 31250,15,625, 7,813 and 3,906 cells). The graph demonstrates that this approach enables the coverage of most lipid classes even in the lower cell dilution. Exceptions make lipid species of the GM3, LPA, LPC-O, LPE-O, LPG, LPI, PG-O, PI-O, PS-O and DGDG subclasses, which are not detectable in negative ion at the low cell number, e.g., 3,906 cells.

The comparative quantification values of lipids obtained across the various dilutions in both positive and negative ion species mode of lipids extracted with the optimized dual extraction method was carried out to determine the lower limit of quantification of lipid classes and/or species in relation to the number of cells extracted. Supplementary Material shows the relative quantification results in negative and positive ion mode using the dual extraction method across the different dilutions. The peak area linearity was calculated for both ion modes, with a R^2^ value superior to 0.8 for 90.51% and 91.11% of the lipids in positive ion mode and negative mode, respectively. Conversely, less abundant lipid species maintain consistent quantification values even at the lowest dilutions. The lowest dilution point corresponds to lipid extracts from 157 cells injected on the column in negative ion mode and 79 in positive ion mode, which substantiates a good sensitivity of the overall workflow. Particularly, considering that no instrumentation specific to single-cell omics was here used approach (Gerichten et al., 2023).

In Figure 5B, the relative quantification values of 9 cell dilutions obtained from the two extraction methods are contrasted. The PE lipid class generally demonstrates higher values with classical extraction than with dual extraction method in positive ion mode, while LPCs, LPEs, and PIs are more prevalent with dual extraction compared to classical extraction in negative ion mode. However, these differences are particularly significant only for specific lipid species within these classes; overall results indicate a similar level of relative quantitation profile under these conditions. The dual extraction method appears comparable to classical extractions’ quantification values and offers an additional advantage by allowing RNA extraction to add a new omic dimension.

**FIGURE 6:**
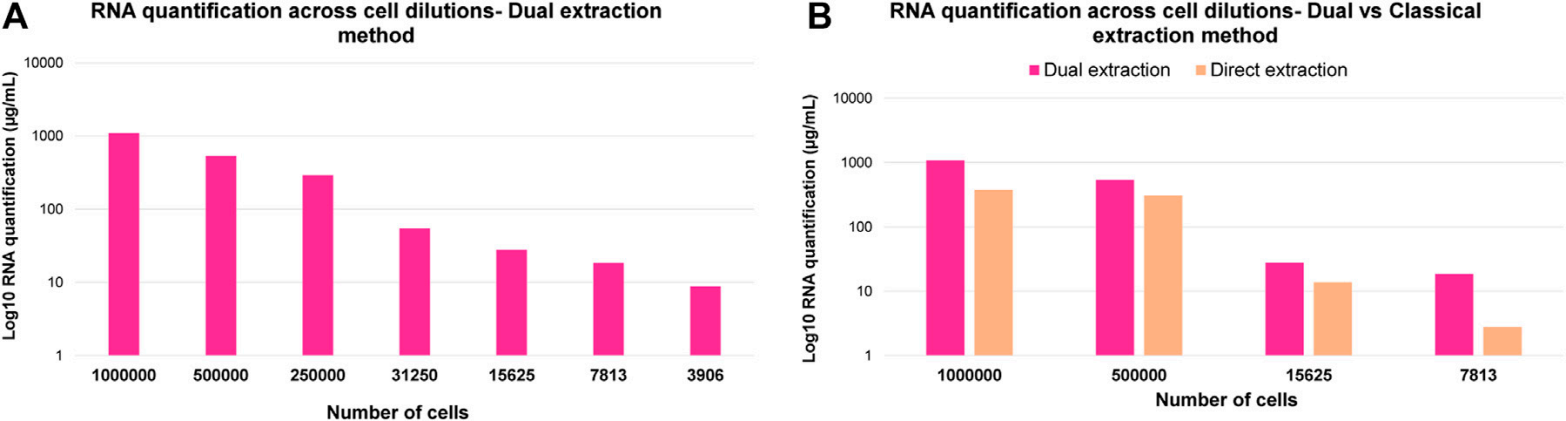
**(A)** Log_10_ RNA quantification results across cell dilutions corresponding to 1,000,000, 500,000, 250,000, 31,250, 15,625, 7,813 and 3,906 cells. **(B)** Comparison of Log_10_ RNA quantification results across cell dilutions corresponding to 1,000,000, 500,000, 15,625 and 7,813 cells after direct and dual extraction.

In order to further demonstrate the suitability of this optimized dual extraction protocol for analyzing both omic layers, lipidomics and transcriptomics, RNA was extracted from the aqueous phase using RNeasy^®^ Micro Kit protocol designed for human cells and subsequently quantified. The RNA was in parallel also extracted and quantified directly from the cell pellet to compare and benchmark the performance of dual lipidome/transcriptome extraction from cells. Figure 6A illustrates how the RNA quantification values decrease linearly across cell dilutions, while Figure 6B demonstrates a significant improvement in RNA extraction yield when using dual extraction compared to direct extraction of RNA. Additionally, it shows that this optimized method is adequate for RT-qPCR analysis with as few as 31,250 cells for 0.5 ug of RNA when utilizing the entire volume of extract. RNAseq can be performed on 7,813 cells with a total RNA of approximately 150 ng, meeting the required amount of genetic material for this analysis. Hence, these findings indicate that the optimized multi-extraction method and the selected kit are appropriate for isolating RNA, making it readily amenable for subsequent analysis using RNA-seq or qPCR, depending on the specific objectives of the study.

To conclude, satisfactory global qualitative and quantitative profiling of lipids and RNAs is obtainable from a low number of cells, using combinatorial approach including: i) co-extraction of lipidome, transcriptome and to some extent metabolome, ii) untargeted 4D-TIMS cellular lipidomic, and iii) transcriptome analysis by RNA sequencing and/or qPCR.

### 3.2 Characterization of MyD88-KO macrophages’ phenotype for atherosclerosis protection: application of optimized multi-extraction for lipids, metabolites, and RNA, as well as 4D-TIMS cellular lipidomics and transcriptomics

In order to showcase the applicability of this cellular multi-omic platform and also to characterize the lipidome and transcriptome associated with an atherosclerosis protective phenotype of macrophages, we investigated the specific molecular changes in MYD88-KO macrophages versus control cells.

To investigate this question, wild type (WT) (C57BL/6J) and knock-out MyD88-KO (C57/129) macrophages from mice were chosen. When MyD88 is knocked out or disabled in macrophages it inhibits the typical signal transduction pathways that would lead to the pro-inflammatory M1 phenotype in macrophages. One critical factor in the initiation and progression of atherosclerosis is the release of inflammatory factors and cytokines produced by the MyD88 upon the activation of toll-like receptor 4 (TLR4). MyD88 contributes to the migration and polarization of macrophages to form M1 macrophages that will release more proinflammatory factors and hence, enhance monocyte migration and plaque formation. Therefore, the key role of MyD88 in the initiation and amplification of this cascade leading to formation and growth of the atherosclerotic plaque, makes it an excellent model to study the impact of reduced pro-inflammatory signaling on the lipidome and transcriptome of macrophages in the context of atherosclerosis. This approach would involve the extraction of lipids, RNA, and metabolites from macrophages.

#### 3.2.1 Macrophages lipidome

**FIGURE 7:**
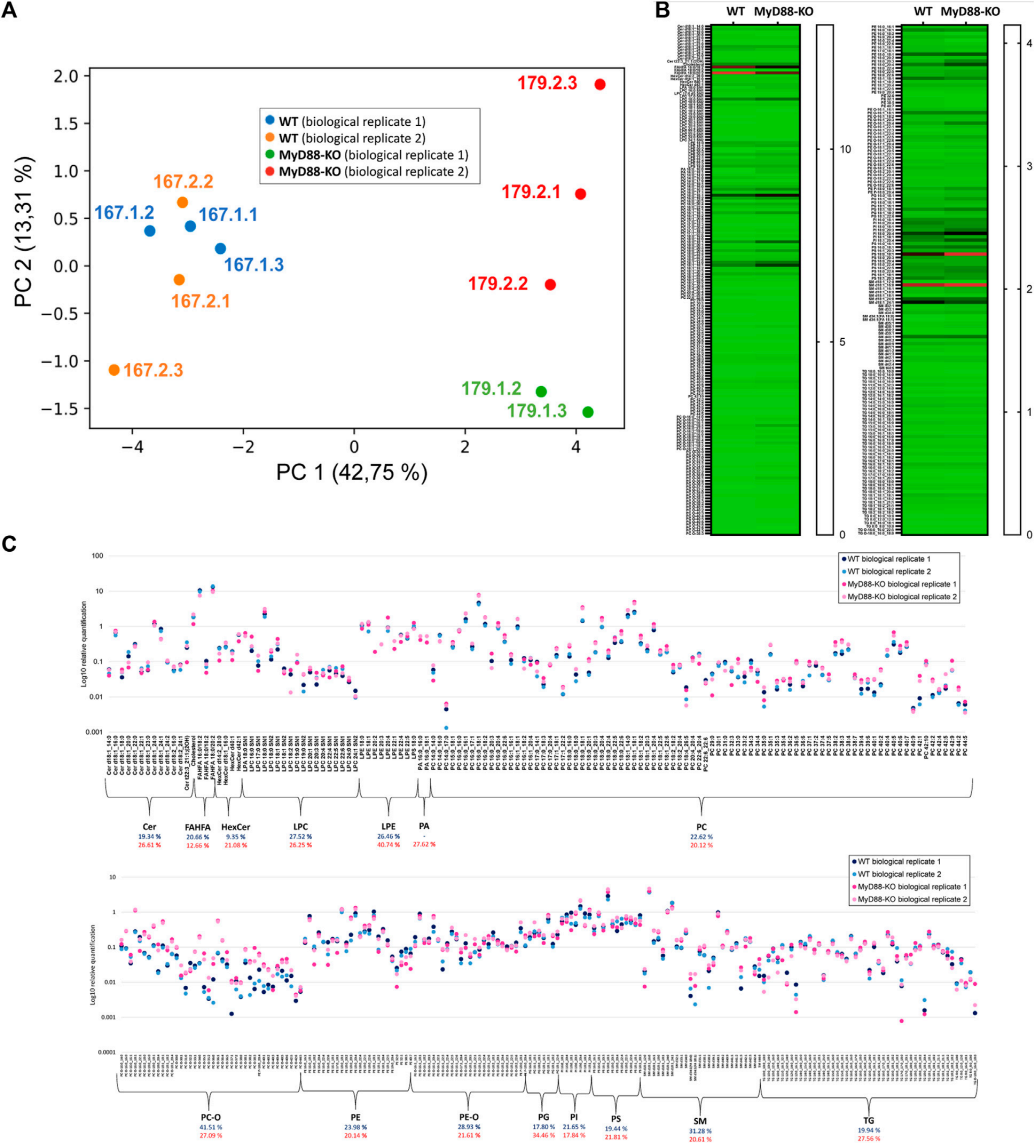
**(A)** PCA of all the quantified lipids using UHPLC (RP)-TIMS-TOF(ESI+) and UHPLC (RP)-TIMS-TOF(ESI-), showing the separation between MyD88-KO and WT macrophages classes. **(B)** Heatmap showing the relative quantification values for lipids comparing two macrophage models (WT and MyD88-KO). **(C)** Scatter plot depicting the logarithmic relative quantification of lipid species and coefficient of variation across lipid classes, with blue representing MyD88-KO and red representing WT macrophages.

This study utilized two biological replicates of MyD88-KO and WT, each consisting of 500,000 cells. To simplify, we labeled the biological replicates of wild-type (WT) and knockout (KO) macrophages as 167.1, 167.2 for WT, and 179.1, 179.2 for KO. The lipidome profiling was performed using three technical replicates for each biological group. The bucket list of lipid signals during data processing in Metaboscape was curated utilizing the previously generated HEK293 4D libraries, “Multi-omic-derived cellular Lipidome 2023 pos” and “Multi-omic-derived cellular Lipidome 2023 neg”, along with other routine databases. Integration of common and specific lipids to both ionization modes in MyD88-KO and WT macrophages was performed for relative quantification. The results were visualized via principal component analysis (PCA) in Figure 7A, where each data point represents one of the technical replicates of both cell types. The loadings for each principal component analysis axis are included in Supplementary Figure S3. Biological replicates of both WT and KO macrophages shows dispersion in the PC2 space due to sample variability. The dispersion is higher in the case of MyD88 macrophages probably due to the heterogeneity of this experimental group triggered by the inactivation of this gene. However, the two macrophage groups are fully distinguishable by their distribution and separation across PC1 space (Figure 7A). One technical replicate “179.1.1” was excluded from the analysis as an outlier due to its noticeably different distribution compared to the other technical replicates in the PCA space. The distinct lipid profile of MyD88-KO macrophages indicates the direct effect of MyD88 on their lipid composition.

The heatmap in Figures 7B, C depicts the relative quantification values of various lipid species in the KO and WT macrophages. While the relative abundance of most FAHFA species is lower in MyD88-KO compared to WT, ether-linked lipids (PC/PE-O) as well as PE and PC, show remarkably higher values for MyD88-KO compared to WT macrophages. The TG species do differ between both cell types. PS, PG and PI phospholipids are also dysregulated in MyD88-KO. Since MyD88 is linked to inflammatory responses, these results underscore the role of lipid patterns in regulating inflammatory pathways associated with atherosclerosis in macrophages (Blagov et al., 2023). This demonstrates the importance of these lipids in the mechanistic pathways that can be potentially involved in regulating anti-inflammatory mechanisms in cardiovascular diseases, which are remarkably important in the formation of the atheroma plaque. The potential of these lipids as important targets for protecting against atherosclerosis, makes the study of their metabolic and mechanistic functions crucial for future research. The reproducibility of relatively quantified levels of lipids and the average coefficient of variation (CV) for each lipid class indicate a good quantitative reproducibility of the MyD88-KO lipidome phenotype. The average CV for both cell types is 21.58% and 22.87% and demonstrate a similar quantitative reproducibility among phenotypes despite the different molecular matrix of both cell types (Chiu et al., 2010). In conclusion, the multi-omic cellular approach used in this study provides a reliable and consistent lipidomic profile of macrophages anti-inflammatory state helping us to derive valuable insights into molecular mechanisms associated with MyD-88 function in macrophages.

**FIGURE 8:**
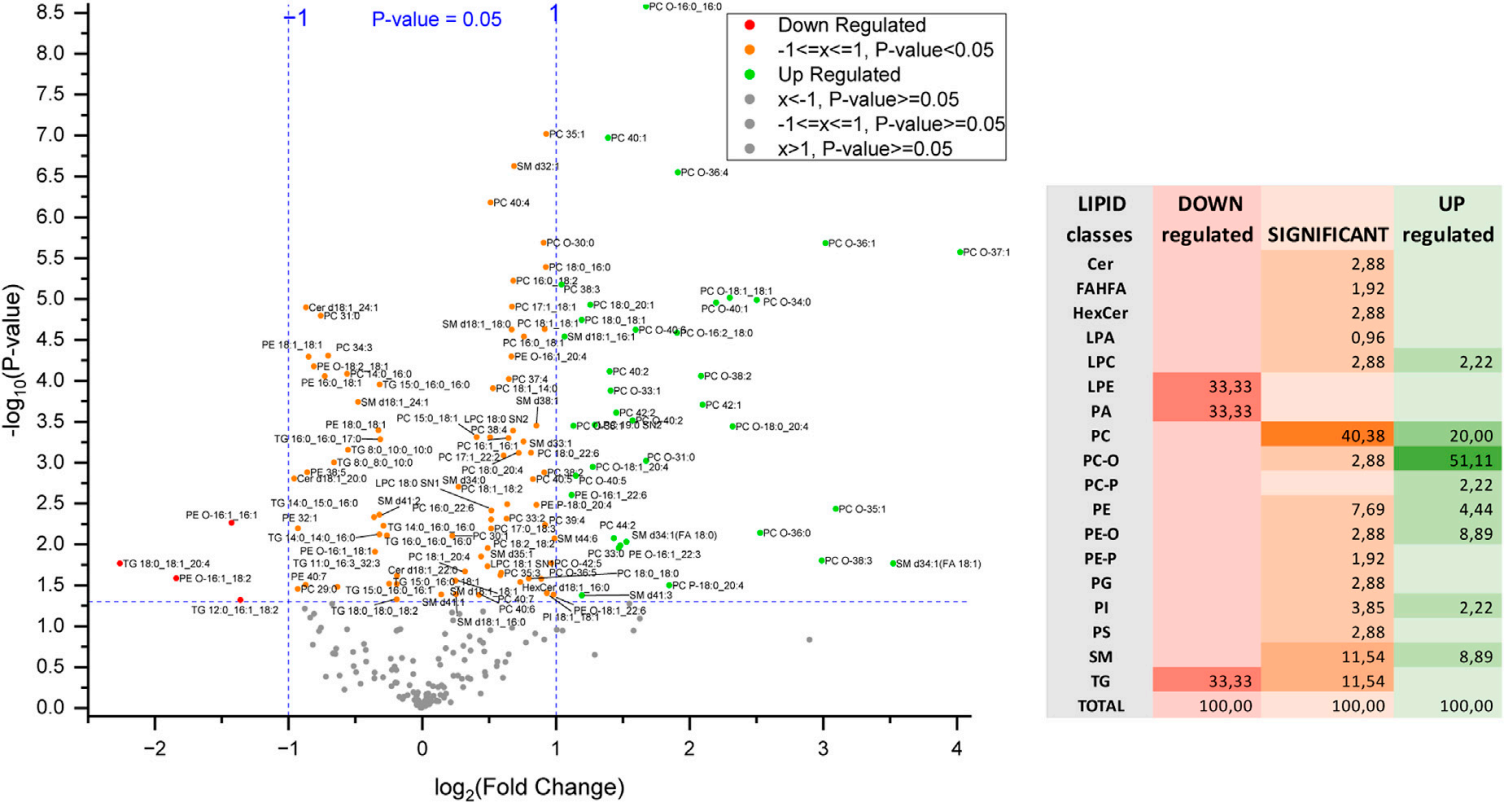
Volcano plot of differentially regulated lipids and percentage contribution of each lipid class to the dysregulation of these lipids in MyD88-KO. Each point represents a lipid species plotted based on fold change and statistical significance. The *x*-axis shows the log_2_-fold change (FC) in lipid expression, with positive values indicating upregulation and negative values indicating downregulation. The *y*-axis represents the -log_10_(*P*-value), reflecting statistical significance. Points above the horizontal threshold line denote lipids with statistically significant changes.

So far, the findings suggest a clear lipid profile difference in the KO cells, potentially linked to the influence of MyD88. To investigate significantly up- or downregulated lipids (*P*-value < 0.05) in this anti-inflammatory macrophage model more closely, a volcano plot was utilized (Figure 8). The percentage contribution of each lipid class to this dysregulation is also visualized. Ether-linked lipids, PC/PE-O and PC/PE-P, constitute the most prominent group of the total upregulated lipids (62.2%) followed by PC and SM. The high representation of the ether-linked lipids and PC lipid class within the upregulated lipids in MyD88-KO macrophages makes them a potential target of the anti-inflammatory mechanism and response. In view of the cell reprogramming events, it will be interesting to explore whether these classes play an essential role in the reprogramming macrophages toward an anti-inflammatory phenotype (Cortés et al., 2023; Kelly and O’Neill, 2015; Pérez and Rius-Pérez, 2022). LPE, TG and PA lipid species contribute equally to the downregulated effect of MyD88 in the lipidome of this macrophage model.

#### 3.2.2 Transcriptomic profiling of WT and MyD88-KO macrophages

RNA obtained via the improved dual-omics extraction technique was analyzed using RNAseq. The RIN are 2.4 for MyD88-KO and 2.3 for WT samples. To facilitate subsequent transcriptome profiling of both WT and MyD88-KO macrophages T, RNAr depletion was carried out. This approach increased specificity in capturing target mRNA molecules by reducing ribosomal RNA presence that could disrupt downstream analysis processes.

**FIGURE 9:**
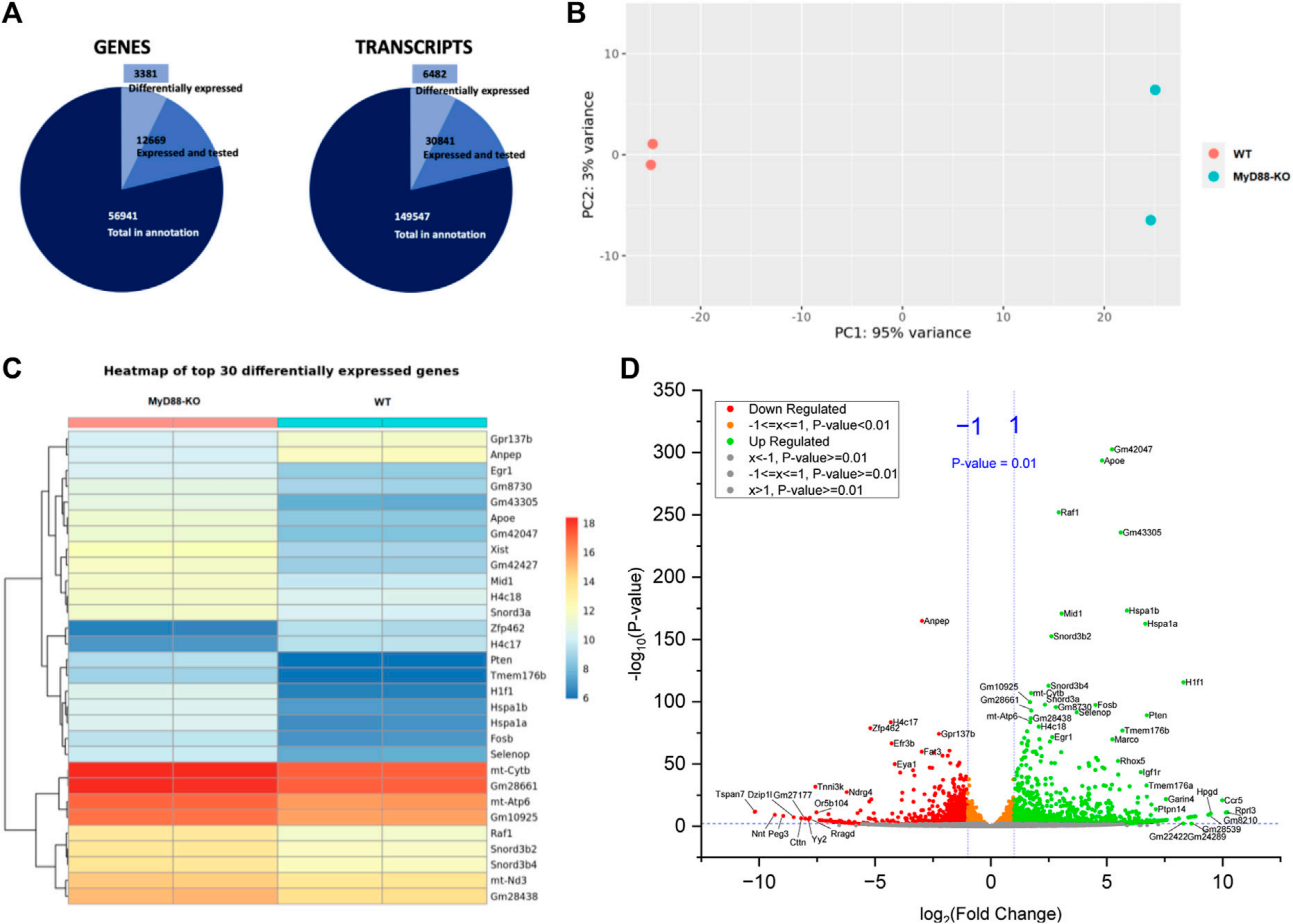
**(A)** Pie chart of genes selected for RNAseq analysis. **(B)** PCA plot of RNAseq sample distribution of WT and KO. **(C)** Heatmap showing the expression of the first 30 differentially expressed genes with adjusted *P*-value < 0.05 for WT and MyD88-KO macrophages. **(D)** Volcano plot of up- and downregulated genes expressed in MyD88. 0.01 was defined as *P*-value threshold as well as a FC of 2.

The pie chart in Figure 9A depicts 3,381 differentially expressed genes and 6,482 differentially expressed transcripts compared to the total count of expressed and tested genes with MYD-88 KO. It provides firsthand information about the extensive high number of genes and transcripts expressed and tested in MyD88, potentially involved in regulating anti-inflammatory pathways modulated by MyD88 in macrophages. Furthermore, Figure 9B shows a PCA plot that illustrate distinct clustering and separation of WT and MyD88-KO macrophages based on their gene expression profiles, with technical replicates of the same cell type clustering together. This group separation fully aligns with the results showed previously in the PCA analysis of the lipidome sample distribution. The genes and transcripts differentially expressed in MyD88-KO compared to WT macrophages primarily contributing to this differentiation are depicted in the heatmap in Figure 9C of the first 30 differentially expressed genes. Selected genes with up- and downregulation trends were based on a *P*-value < 0.01 and fold change (>1 or < -1). Figure 9D illustrates the direction of regulation trends in MyD88-KO macrophages using a volcano plot, displaying the gene expression difference between MyD88-KO and WT macrophages with the significance (*P*-value < 0.01) and a log 2-fold change. The analysis also indicates distinct transcriptome profile differences in this anti-inflammatory macrophage model.

#### 3.2.3 Pathway investigation of MyD88-KO macrophages

**FIGURE 10:**
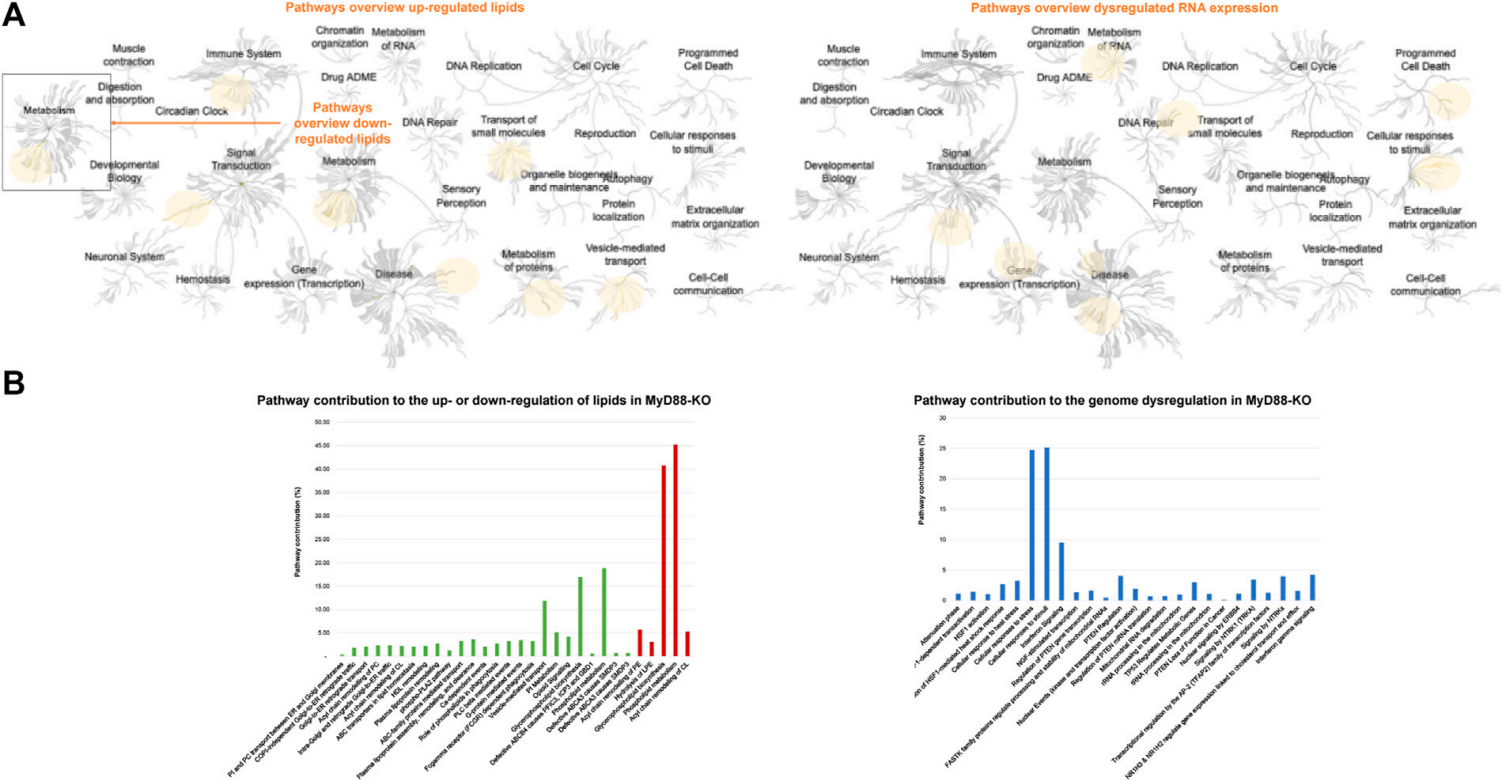
**(A)** General pathways’ overview of up- and downregulated lipids, generated by Reactome (left). General pathways’ overview, generated by Reactome, affected by the first 30 differentially expressed genes with a 99% confidence and fold change superior to 2 (right). **(B)** Contribution of each pathway to the up- or downregulation of lipids in MyD88-KO. Pathway list generated using Reactome. In green those pathways found for upregulated lipids and in red those discovered for the downregulated lipids (left). Contribution of each pathway to the genome dysregulation in MyD88-KO. Pathway list generated using Reactome (right).

Significantly represented lipids and RNA from the globally profiled lipidome and transcriptome in MyD88-KO macrophages compared to WT macrophages, were investigated by Reactome pathway to unveil their mechanistic function in this anti-inflammatory response. The pathway clusters marked in yellow in Figure 10 are those affected by the dysregulation of RNA and lipids in MyD88-KO macrophages. Upon initial examination of these pathways obtained by the comprehensive analysis of the impact of MyD88 deficiency on the transcriptome, there is a significant influence on genes associated with cellular responses to stimuli and signal transduction. The metabolism of RNA, DNA repair, gene expression transcription, and programmed cell death are also impacted, as well as some disease pathways related to cellular responses to stress, DNA repair, transmembrane transporters, signal transduction by growth factor receptors and second messengers and programmed cell death (from lower to higher FDR). Both up- and downregulated lipids impact metabolism, whereby lipid metabolism is predominantly upregulated with only a few downregulated species. An in-depth analysis of the pathways affected by imbalanced lipid levels indicates that both up- and downregulated lipids impact metabolism. Additionally, vesicle-mediated transport, small molecule transport, protein metabolism, immune system function, and disease pathways are specifically affected by the upregulated lipids produced by MyD88 absence. The predominant upregulation of PC-O among the upregulated lipids, substantially influences the dysregulation of the highlighted pathways in Figure 10A, potentially involving their anti-inflammatory effects. The graph in Figure 10B depicts the 25 most significant pathways underlined by the upregulated lipids, and the 5 significantly identified pathways for the downregulated lipids along with their percentage contribution. Only the pathways with a *P*-value < 0.05 and a false discovery rate (FDR) < 0.2 were chosen for further analysis, prioritizing those with the highest significance and lowest FDR. Similarly, to simplify the analysis of the transcriptome, only the first 30 differentially expressed genes between MyD88-KO and WT were selected for pathway discovery based on their *P*-value < 0.01 and fold change > 1 or < −1 (Figure 10B). In both graphs, the pathways with highest significance values are represented from top to bottom in both graphs. These findings indicate that vesicle-mediated transport and glycerophospholipid biosynthesis exhibit the highest contribution rates in this anti-inflammatory macrophage model. In addition, the highest significantly disrupted pathways by the change in the lipidome are related with the membrane transport and trafficking of molecules between Golgi and endoplasmic reticulum (ER), as well as the remodeling of other lipids and proteins such as: PC, cardiolipins (CL), high density lipoprotein (HDL) and other plasma lipoproteins. Other pathways affected are ATP-binding cassette (ABC) transporters and G-proteins transport, phospholipase C beta (PLCβ) and A2 and (PLA2) metabolism and phagocytosis mediated by phospholipids and by Fc gamma receptor (FCGR). The pathways affected by the transcriptome disruption, cellular response to stress and stimuli as well as interferon (IFN) signaling pathways, have the highest contribution rates and are also the most significant in this anti-inflammatory macrophage model.

The mechanistic function of these pathways is potentially involved in anti-inflammatory protection against the development of macrophages’ pathogenic phenotypes. Thus, remodeling of these pathways also by alternative, or adjuvant venues to knocking out the MyD88 could play a crucial role in the re-programming of macrophages to prevent the formation of atheroma plaques and advancement of atherosclerosis disease.

## 4 Discussion

The suitability of 4D-cellular multi-omics for analyzing cellular lipids and metabolites, from single lipidome extraction or dual extraction of lipids and RNA stemming from a small number of cells was demonstrated using HEK293 as a cell model. In terms of lipids and RNA cellular resolution, the qualitative and relative quantitative analysis of the lipidome and the relative quantitative analysis of the total extracted RNA of the serial dilutions of cell numbers, illustrated the applicability range of this protocol when working with minimal sample amounts. Similar or increased levels of certain lipid classes, such as PI, SM, PE-O/P and LPE was obtained by dual lipid and RNA extraction compared to lipid-only extraction protocol, suggesting a differential dislocation of these lipids from the cell’s biomolecular network due to physico-chemical properties these extraction parameters are enabling. A core set of cca 100 lipids mostly covering the glycerophospholipidome are consistently detected and can be quantified in high (cca 1 mil) and lower number cells (cca 3,900). Determination of the lower limit of quantification and detection, in terms of the lowest number of cells required for detection of individual cellular lipid species is valuable in deriving the lipid function knowledge that is preserved with lowering number of cells. Besides, it also allows prospective informed-study design, including selection of minimum number of cells for a given set of lipid targets, when aiming to investigate specific lipid alteration in cellular populations.

This is particularly advantageous when dealing with limited sample availability. This comprehensive dual extraction method combined with 4D-TIMS cellular lipidomics and RNA analysis is essential for protecting and maximally utilizing valuable scarce samples as well as for improving molecular read-out/per sample. It also reduces errors from single-variable data variability and diverse molecular distribution characteristics, during aliquoting and sampling facilitating effective data combination. Certainly, combining the dual extraction protocol with analytical technologies dedicated to single-cell omic analysis, single cell lipidomics and transcriptomics is expected to increase the lipidome and transcriptome coverage and depth of functional analysis in cells. Moreover, prospective inclusion of metabolite analysis will add a new molecular dimension, making this approach an excellent tool for translational biology. (Chen et al., 2023; Bayer and Alcaide, 2021).

The effects of MyD88 in the immune system activity and its correlation with some disease processes are rather well studied, particularly its high potential as a target for combating inflammatory processes involving important pathways such as NF-kB and AP-1. As an example, a recent study demonstrated that MyD88 deletion decreased macrophage recruitment and affected macrophage function in plaques (Bayer and Alcaide, 2021). Macrophages isolated from MyD88^−/−^ mice exhibited reduced activation, lipid accumulation and foam cell formation in response to ox-LDL treatment, a key factor in atherosclerosis. Additionally, endothelial reactive oxygen species formation, which drives ox-LDL formation, was also decreased (Bayer and Alcaide, 2021). Therefore, the reasons why MyD88 antagonists were largely studied to combat inflammation and associated diseases such as atherosclerosis are evident. Small molecules that mimic the BB-loop in the Toll/IL-1 receptor (TIR) domain of MyD88 were found to inhibit MyD88-mediated pro-inflammatory signaling (Saikh, 2021). Clinical trials using MyD88-targeted therapy for chronic obstructive pulmonary diseases have shown promising results. Dietary supplementation with glycosaminoglycans such as chondroitin sulfate has also been observed to inhibit MyD88-dependent inflammatory signaling in chondrocytes. Medications in the tricyclic family targeting neurotransmitter release and uptake, as well as opioids, have been shown to modulate TLR activity and MyD88 activity respectively (Bayer and Alcaide, 2021). These results reinvigorated the idea that MyD88-targeted therapeutic intervention of pro-inflammatory signaling could be feasible in attenuating severe inflammatory diseases and opens a great opportunity in treating chronic inflammatory diseases. All of these drugs showed varying levels of activity on TLR/MyD88 signalling, and have varying pharmacodynamic properties, therefore could be useful for specific forms of cardiovascular diseases (CVD) depending on the exact contributions of MyD88. However, while safety profiles have already been established, further study would need to be done to demonstrate utility in repurposing them for CVD, since MyD88 antagonists may also impact the protective mechanisms against macrophage infections.

Therefore, it is crucial to thoroughly study the molecular targets for addressing chronic diseases due to the influence of MyD88 cascades on TLR4 signal activation, which significantly contributes to inducing trained immunity (Owen et al., 2022). TLR/MyD88 signalling extends beyond immune cells, and most of the work in preclinical models have used globally deficient mice. However, upregulated TLR/MyD88 signalling has been shown to alter endothelial cell function and contribute to the pathogenesis of vascular disease, expanding the importance of this pathway to other cardiovascular cell types (Guerrini and Gennaro, 2019).

For this reason, the lipidome- and transcriptome-associated anti-inflammatory cascade and the implied partners were studied in this study in the search of good alternatives in the protective mechanism against atherosclerosis in macrophages.

Multi-omics studies involve large and complex datasets. To simplify the functional analysis of RNA and lipids in context of MyD88 depletion, we used two different software tools, LIPEA and Reactome, to filter and visualize the most significant results and focused here our further discussion. on the multi-omic profile of a macrophage model in an anti-inflammatory state for atherosclerosis protection. When looking at each molecular layer independently, i.e., the lipidome dysregulation in MyD88 macrophages, the predominant role of plasmalogens upregulation in this anti-inflammatory response is evident. Ether-linked lipids (PC/PE-O) contribute 60% of the total upregulated lipids constituting the most prominent group. Plasmalogens are unique membrane glycerophospholipids with a vinyl-ether bond at the sn-1 position and enriched in polyunsaturated fatty acids at the sn-2 position. Their physiological roles vary across different tissues, metabolic processes, and developmental stages due to their lability to oxidation and utilization by higher organisms (Braverman and Ann, 2012). The high representation of this lipid class within the upregulated lipids in MyD88-KO macrophages makes them a potential target of the anti-inflammatory mechanism and response. The correlation between plasmalogens and atherosclerosis can be understood in terms of the role plasmalogens play in cellular functions and oxidative stress, whereby antioxidant properties can protect cells from oxidative stress by scavenging reactive oxygen species. Plasmalogens also play a role in anti-inflammatory mechanisms, being present in inflammatory cells are believed to regulate the function of enzymes associated with inflammation. Lower levels of plasmalogens have been associated with multiple inflammation diseases. including in atherosclerosis (Ridker and Thomas, 2014; Wallner et al., 2018). As they are present in endothelial cells, and endothelial dysfunction is an early event in atherosclerosis, changes in their levels might impact endothelial function, which plays a pivotal role in maintaining vascular health. Low plasmalogen levels have been found in individuals with atherosclerosis, suggesting a potential role for plasmalogens in the development or progression of the disease. However, while there are strong associations between plasmalogen levels and atherosclerosis, the exact mechanistic links are not fully defined and further research is required to fully elucidate the precise role plasmalogens play in atherosclerosis and whether modulation of plasmalogen levels could be used as a therapeutic strategy for cardiometabolic diseases, including atherosclerosis (Paul et al., 2019; Braverman and Ann, 2012; Deng and Angelova, 2021).

Studies carried out until the moment, have focus their attention in the influence of dietary changes through increasing plasmalogens levels in inflammation and disease as well as the *in vitro* effects of changes in the lipid profile in plasmalogens levels. As an example, Wallner et al., 2018 *in vitro* showed the effects of oxidized lipoproteins in plasmalogen levels in human monocyte derived macrophages (Wallner et al., 2018; Paul et al., 2019 demonstrates that phagocytic activity of plasmalogen deficient mutant macrophages was significantly improved when plasmalogen content was restored through supplementation of lysoplasmalogen, highlighting the importance of plasmalogens in regulating macrophage phagocytic activity (Paul et al., 2019). Plasmalogen enrichment via batyl alcohol supplementation attenuated atherosclerosis in ApoE- and ApoE/GPx1-deficient mice (Rasmiena et al., 2015). Lin D. and collaborators also demonstrated that the supplementation with DHA/EPA enriched ethanolamine plasmalogen (EPA-PlsEtn) dramatically reduced atherosclerotic lesions by 78% (Ding et al., 2020). A reduction in arachidonate-containing plasmalogens has been noted in extensively diseased carotid plaque samples compared to minimally diseased ones (Ménégaut et al., 2019). In this context, our findings of upregulated plasmalogens in MyD88 KO macrophages concur well with the above previous findings on their role as anti-inflammatory regulators. It would be interesting to prospectively investigate whether targeted increase of plasmalogens will suffice acquisition of an anti-inflammatory macrophage phenotype (that is without MyD-88 depletion), and also generally how cell-reprogramming into therapeutic functions can be achieved by modulating specific lipid content.

The pathway analysis determination of the transcriptome has rendered important data regarding the potential disrupted pathways during this inflammatory protective model, such as cellular response to stimuli and stress and interferon signaling. From all the significantly dysregulated genes in this model apolipoprotein E (ApoE) is highlighted due to its role in lipid metabolism, and its implications in inflammation, particularly in the context of atherosclerosis. ApoE is 1 out of 5 of the most significant genes, out of the total 3,381 differentially expressed in this model. APoE affects 3 of the 25 significantly identified pathways which are: nuclear signaling by ERBB4, transcriptional regulation by the AP-2 (TFAP2) family of transcription factors and NR1H3 & NR1H2 regulate gene expression linked to cholesterol transport and efflux. ApoE is involved in clearing lipoproteins and cholesterol efflux, potentially reducing the opportunity for foam cell formation and subsequent inflammation contributing to atherogenesis. ApoE’s anti-inflammatory properties might also influence the stability of atherosclerotic plaques. A deficiency in ApoE can lead to increased inflammation and oxidative stress, contributing to plaque instability such as inhibiting T cell proliferation and dampening cytokine production by macrophages (Yamazaki et al., 2019). However, different apoE alleles can have distinct effects. Genetic variation in ApoE can affect atherosclerosis risk. The ε4 variant has been associated with higher cholesterol levels and increased risk for cardiovascular disease, whereas the ε2 variant is often associated with lower risk (Ringman et al., 2014). ApoE is significantly overexpressed in MyD88-KO macrophages with a *P*-value of 3.07 × 10^−294^ and a FC of 4.8 (Supplementary Material). Overall, the correlation between ApoE and atherosclerosis is well-established, with the protein playing a multifaceted role in lipid metabolism, inflammation, and atherogenesis. The presence and function of ApoE are thus critical factors in the overall risk and development of atherosclerotic disease. Due to the expression complexity of this gene, further studies will focus on the identification of the corresponding alleles.

Multi-omics can provide valuable insights into the lipidome alterations occurring together with gene expression patterns in MyD88-KO macrophages during the anti-inflammatory state, shedding light on the underlying mechanisms involved in atherosclerosis protection. Noteworthy here is that the primary lipid changes, (PCs, PC-Os and PE-Os) in MyD88-KO uniquely underscore pathways of immune system, transport of small molecules, vesicle-mediated transport and metabolism of proteins. Complementary, cell-survival and death, cellular response, and metabolism of RNA and transcription are uniquely delineated by gene expression changes. This newly provided knowledge on the specific function of plasmalogens in mediating immune response and metabolism of proteins in anti-inflammatory macrophages is of general importance to understand macrophage reprogramming and strategies to drive this process for therapeutic purpose. In addition, new strategies in multi-omic studies at high-cell resolution are of vital importance to provide new insights in disease mechanisms in limited samples and tissue microenvironments. Low-cell number multi-omic studies present challenges in relation to sensitivity. However, this new approach presents a coverage of 89 lipids in the lowest cell dilution analyzed using the dual extraction protocol in HEK293 cells, corresponding to lipids injected from 157 cells in negative ion mode and 79 cells in positive ion mode. Even though these results can vary depending on the studied cell type, as demonstrated in other studies, the results highlight the sensitivity of this new approach (Gerichten et al., 2023).

It is conceivable that proteins and other water-soluble molecules can be extracted from the aqueous flow-through remaining after RNA extraction, following optimisation based on the existing micro RNEasy QIAGEN protocols (Micro Handbook, 2021). Given that, essentially, the micro RNEasy extraction protocol was only modified by the addition of chloroform, subsequent protein purification, buffer exchange of the aqueous phase and optimization of protein extraction are envisaged to enable also efficient proteome extraction in combination with dual extraction of lipids and RNA. Also, considering the advents in single cell proteomics, the proteome analysis following such an integrated lipidome/transcriptome/proteome analysis is envisaged to be feasible. We expect that the future combination of the extraction of proteins and other metabolites alongside the dual extraction and analysis of RNA and lipids, coupled with high-end proteomics, metabolomics, lipidomics and transcriptomics, will enable the prediction of disease biomarkers and potential therapeutic targets (Åkesson et al., 2023).

Overall, this model for atherosclerosis protection can further enhance our understanding of the specific role of MyD88 signaling in the development and progression of atherosclerosis. Through this multi-omic cellular approach, researchers can elucidate the molecular pathways and regulatory networks involved in the anti-inflammatory state of macrophages and identify novel targets and biomarkers for the diagnosis, treatment, and prevention of atherosclerosis. Enhancing plasmalogen levels could possibly offer a promising and safe therapeutic approach for mitigating atherosclerosis and lowering cardiovascular disease risk, especially in situations characterized by increased oxidative stress and inflammation. Future studies will be focused on the multi-facetted role of ApoE and prospective analysis of plasmalogens causal relationship and potential therapeutic implications of the macrophages’ protective profile against atherosclerosis.

## Data Availability

The transcriptomic data presented in the study are deposited in the NCBI repository (https://www.ncbi.nlm.nih.gov/bioproject), accession number PRJNA1148506. Lipidomic data are available in the Supplementary Material.
