# Supplementary material for: Integrated cellular 4D-TIMS lipidomics and transcriptomics for characterization of anti-inflammatory and anti-atherosclerotic phenotype of MyD88-KO macrophages
Source: Front Cell Dev Biol. 2024 Aug 23;12:1450971. doi: 10.3389/fcell.2024.1450971 (PMC11377276; doi:10.3389/fcell.2024.1450971)
Supplement: Supplementary file 1 [file Table1.DOCX]

Supplementary Material

**Integrated cellular 4D-tims lipidomics and transcriptomics for characterization of anti-inflammatory and anti-atherosclerotic phenotype in MyD88-KO macrophages**

# Supplementary Data

**Supplementary Data 1: Lipid deuterated and non-deuterated internal standards (2.2.1.):**

1-pentadecanoyl-2- oleoyl(d7)-sn-glycero-3-phosphate (PA-d7-15:0/18:1), 1-heptadecanoyl-2-(9Z-tetradecenoyl)-sn-glycero-3-phosphocholine (PC 17:0/14:1), 1-heptadecanoyl-2-(9Z-tetradecenoyl)-sn-glycero-3-phosphoethanolamine (PE 17:0/14:1), 1-heptadecanoyl-2-(9Z-tetradecenoyl)-sn-glycero-3-phosphoglycerol (PG 17:0/14:1), 1-heptadecanoyl-2-(9Z-tetra-decenoyl)-sn-glycero-3-phosphoinositol (PI 17:0/14:1), 1-heptadecanoyl-2-(9Z-tetradecenoyl)-sn-glycero-3-phosphoserine (PS 17:0/14:1), cholest-5-en-3β-ol(d7) (cholesterol d7), 1-heptadecanoylglycero-3-phosphate (LPA 17:0), 1-(10Z-heptadecenoyl)-sn-glycero-3-phosphocholine (LPC 17:1), 1-(9Z-heptadecenoyl)-sn-glycero-3-phosphoethanolamine (LPE 17:1), N-palmitoyl-D-erythrosphingosine (d7) (Cer d18:1-d7/16:0), (N-oleoyl-D-erythrosphingosyl-phosphorylcholine-d9 ((SM d18:1/18:1)-d9), 5-[((13,13,14,14,15,15,16,16,16-d9)palmitoyl)hydroxy]-stearic acid (FAHFA 16:0/18:0 d9), 1-heptadecanoyl-2-hydroxy-sn-glycero(d5)-3-phosphocholine (LPC 17:0 d5), 1- heptadecanoyl-2-oleoyl-sn-glycero(d5)-3-phosphocholine (PC 17:0/ 18:1 d5), 1-heptadecanoyl-2-hydroxy-sn-glycero(d5)-3-phosphoethanolamine (LPE 17:0 d5), 1-heptadecanoyl-2-myristoleoyl-sn-glycero(d5)-3-phosphoethanolamine (PE 17:0/14:1 d5), 1-heptadecanoyl-2-myristoleoyl-sn-glycero(d5)-3-phosphoinositol (ammonium salt) (PI 17:0/14:1 d5), 1-heptadecanoyl-2-myristoleoyl-sn-glycero(d5)-3-phospho-L-serine (sodium salt) (PS 17:0/14:1 d5), 1- heptadecanoyl-2-oleoyl-sn-glycero(d5)-3-phospho-(1’-rac-glycerol) (sodium salt) (PG 17:0/18:1 d5), 1,3(d5)-ditetradecanoyl-2-(9Z-hexadecenoyl)-glycerol (TG 14:0/16:1/14:0 d5), were also obtained from Merck (Avanti Polar Lipids, Inc., USA).

# Supplementary Figures and Tables

## Supplementary Figures

**Supplementary Figure 1.** **A.** Lipid quantification comparison of 3 extraction solvents (A, B and C) using dual method in ESI+. Log_10_ of the quantiﬁed values was used for the visualization. Color grade from dark green to light yellow indicating each lipid specie abundance. **B.** Lipid quantification comparison of 3 extraction solvents (A, B and C) using dual method in ESI-. Log_10_ of the quantiﬁed values was used for the visualization. Color grade from dark green to light yellow indicating each lipid specie abundance. **C.** Lipid quantification comparison of 3 extraction solvents (A, B and C) using classical method in ESI+. Log_10_ of the quantiﬁed values was used for the visualization. Color grade from dark to light blue indicating each lipid specie abundance. **D.** Lipid quantification comparison of 3 extraction solvents (A, B and C) using classical method in ESI-. Log_10_ of the quantiﬁed values was used for the visualization. Color grade from dark to light blue indicating each lipid specie abundance.


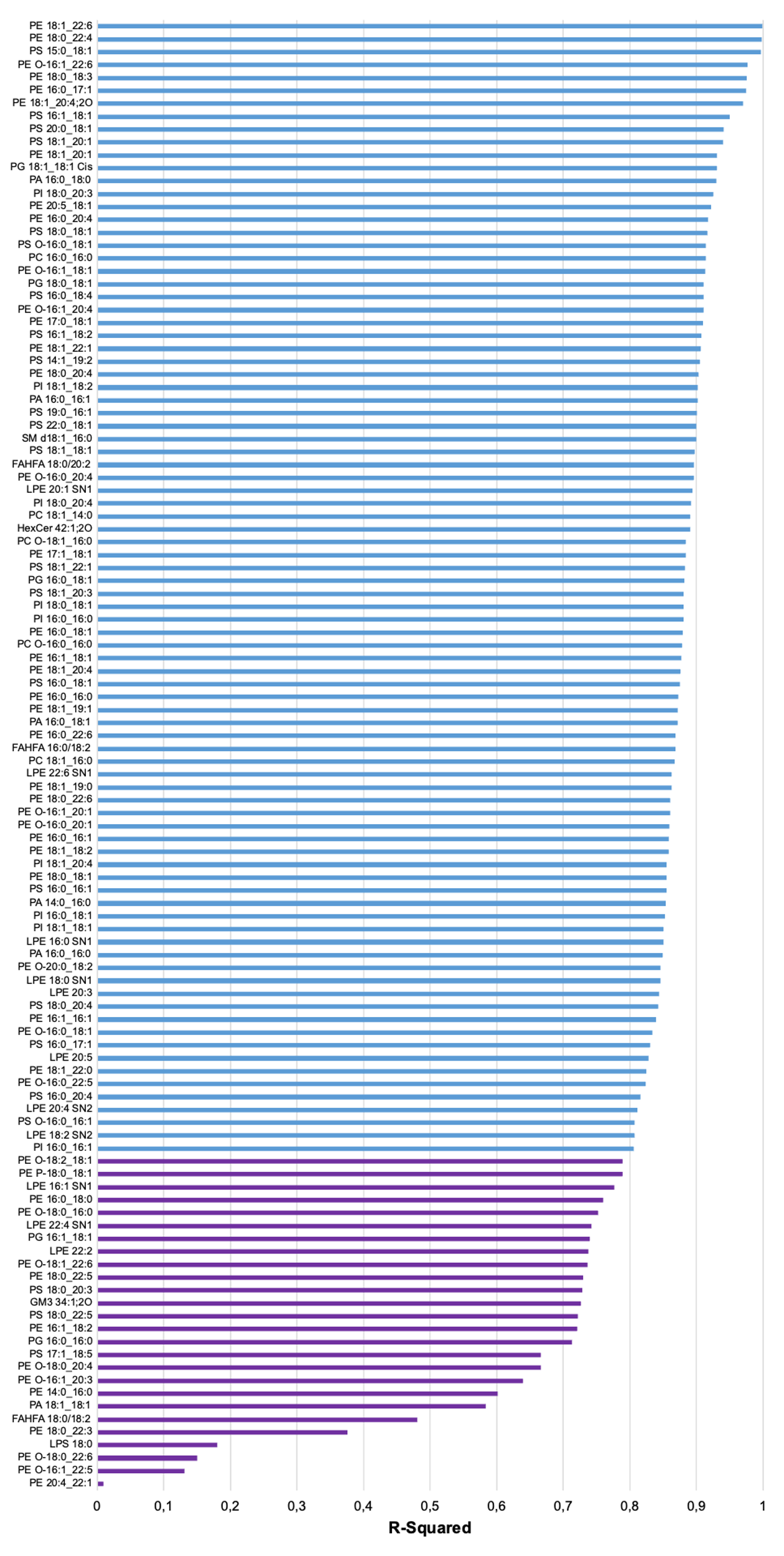


**Supplementary Figure 2.** Peak area linearity fit for the lipid species identified in 85713, 28571, 14285, 7141 and 3572 cells’ organic extracts with a R^2^ value ranging from 0.8 to 1 in ESI-.

**Supplementary Figure 3.** PC1 (left) and PC2 (right) of the PCA loadings of the analysis of all the quantified lipids using UHPLC(RP)-TIMS-QTOF(ESI+) and (ESI-).
